# Lowering Low-Density Lipoprotein Particles in Plasma Using Dextran Sulphate Co-Precipitates Procoagulant Extracellular Vesicles

**DOI:** 10.3390/ijms19010094

**Published:** 2017-12-29

**Authors:** Jiong-Wei Wang, Ya-Nan Zhang, Siu Kwan Sze, Sander M. van de Weg, Flora Vernooij, Arjan H. Schoneveld, Sock-Hwee Tan, Henri H. Versteeg, Leo Timmers, Carolyn S. P. Lam, Dominique P. V. de Kleijn

**Affiliations:** 1 Department of Surgery, Yong Loo Lin School of Medicine, National University of Singapore, 119228 Singapore, Singapore; A0095171@u.nus.edu (Y.-N.Z.); floravernooij@hotmail.com (F.V.); 2 Cardiovascular Research Institute, National University Heart Centre Singapore, 117599 Singapore, Singapore; 3 Department of Physiology, Yong Loo Lin School of Medicine, National University of Singapore, 117593 Singapore, Singapore; 4 School of Biological Sciences, Nanyang Technological University, 637551 Singapore, Singapore; SKSze@ntu.edu.sg; 5 Experimental Cardiology Laboratory, University Medical Center Utrecht, 3584 CX Utrecht, The Netherlands; S.vandeWeg@umcutrecht.nl (S.M.v.d.W.); A.Schoneveld@umcutrecht.nl (A.H.S.); 6 Department of Medicine, National University of Singapore, 117599 Singapore, Singapore; mdctshw@nus.edu.sg; 7 Einthoven Laboratory for Experimental Vascular Medicine, Leiden University Medical Centre, 2333 ZA Leiden, The Netherlands; h.h.versteeg@lumc.nl; 8 Department of Cardiology, University Medical Center Utrecht, 3584 CX Utrecht, The Netherlands; L.Timmers@umcutrecht.nl; 9 National Heart Centre Singapore, Duke-NUS Graduate Medical School, 169857 Singapore, Singapore; carolyn.lam@duke-nus.edu.sg; 10 Department of Cardiology, University Medical Center Groningen, 9713 GZ Groningen, The Netherlands; 11 Department of Vascular Surgery, University Medical Center Utrecht, 3584 CX Utrecht, The Netherlands; 12 Netherlands Heart Institute, 3511 EP Utrecht, The Netherlands

**Keywords:** extracellular vesicles, lipoprotein particles, low-density lipoprotein, LDL, LDL apheresis, coagulation, fibrinolysis

## Abstract

Plasma extracellular vesicles (EVs) are lipid membrane vesicles involved in several biological processes including coagulation. Both coagulation and lipid metabolism are strongly associated with cardiovascular events. Lowering very-low- and low-density lipoprotein ((V)LDL) particles via dextran sulphate LDL apheresis also removes coagulation proteins. It remains unknown, however, how coagulation proteins are removed in apheresis. We hypothesize that plasma EVs that contain high levels of coagulation proteins are concomitantly removed with (V)LDL particles by dextran sulphate apheresis. For this, we precipitated (V)LDL particles from human plasma with dextran sulphate and analyzed the abundance of coagulation proteins and EVs in the precipitate. Coagulation pathway proteins, as demonstrated by proteomics and a bead-based immunoassay, were over-represented in the (V)LDL precipitate. In this precipitate, both bilayer EVs and monolayer (V)LDL particles were observed by electron microscopy. Separation of EVs from (V)LDL particles using density gradient centrifugation revealed that almost all coagulation proteins were present in the EVs and not in the (V)LDL particles. These EVs also showed a strong procoagulant activity. Our study suggests that dextran sulphate used in LDL apheresis may remove procoagulant EVs concomitantly with (V)LDL particles, leading to a loss of coagulation proteins from the blood.

## 1. Introduction

Circulating extracellular vesicles (EVs) have been recognized for their emerging roles in (patho)physiology and their potential as biomarkers in cardiovascular disease [1,2]. We have shown that cardiovascular events, such as myocardial infarction and stroke, are associated with coagulation-pathway proteins that are carried by the plasma EVs [3,4]. Tissue factor, which plays a central role in thrombus formation, is also carried in the blood by EVs [5,6,7]. Furthermore, the concentration of plasma EVs is positively correlated with total thrombin generation in individuals with familial hypercholesterolemia [8].

High levels of low-density lipoprotein (LDL) and very-low-density lipoprotein (VLDL) cholesterol are associated with an increased risk of cardiovascular events [9,10]. For most hypercholesterolemic patients, a standard diet and lipid-lowering drug therapy are recommended. However, patients with severe hypercholesterolemia, such as homozygous familial hypercholesterolemia, may require additional treatment, such as LDL apheresis, a procedure that eliminates LDL and VLDL from the blood [11,12,13,14,15]. One of the most commonly used methods, dextran sulphate adsorption (DSA), has been reported to deplete blood coagulation factors [16,17,18,19]. Furthermore, it was shown that both the extrinsic and intrinsic coagulation pathways were disturbed by LDL apheresis [18]. Depletion of coagulation factors by apheresis is assumed to reduce blood viscosity and improve regional myocardial perfusion in patients with severe hypercholesterolemia [20,21].

Connolly and colleagues showed that the concentration of plasma EVs in patients with familial hypercholesterolemia was reduced after LDL apheresis [8]. The content of these apheresis-depleted plasma EVs, however, has not been characterized. Therefore, it remains unclear if coagulation proteins are present in the plasma EVs and whether these EVs have coagulation activity. We hypothesize that besides (V)LDL, a subset of plasma EVs containing high levels of coagulation proteins will be removed from the blood during DSA LDL apheresis. In this study, we precipitated (V)LDL particles from human plasma using dextran sulphate (as an in vitro model for DSA apheresis) followed by a proteomic analysis of the content and a measurement of the pro-coagulant activity of precipitated EVs.

## 2. Results

### 2.1. Coagulation Protein Levels in the (V)LDL (Very-Low- and Low-Density Lipoprotein) Precipitate

Label-free proteomics was performed on both (V)LDL- and high-density lipoprotein (HDL)-precipitates. Protein levels in the HDL-precipitate were used as reference for proteomic analysis. The proteomic analysis showed that 144 proteins were exclusively present in the (V)LDL precipitate (Supplementary Table S1). Using Ingenuity Pathway Analysis (IPA), three canonical pathways were over-represented in the (V)LDL precipitate: coagulation system, extrinsic thrombin activation, and intrinsic thrombin activation (Table 1).

**Figure 1:**
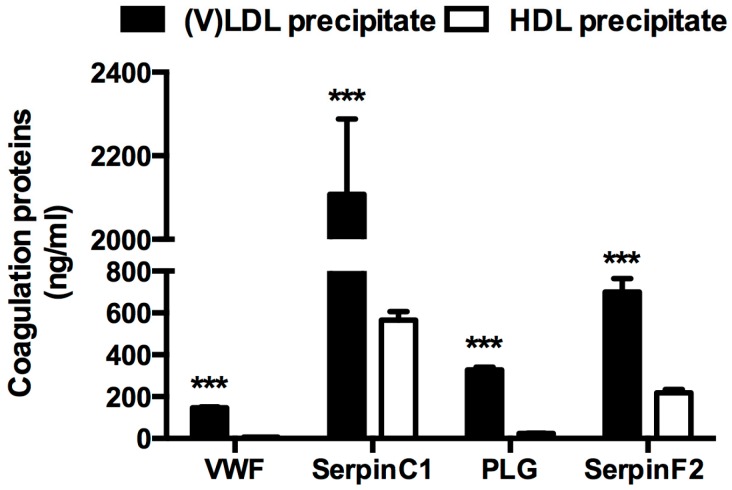
Coagulation proteins enriched in the very-low- and low-density lipoprotein ((V)LDL) and high-density lipoprotein (HDL) precipitates. von Willebrand factor (VWF), SerpinC1, plasminogen (PLG) and SerpinF2 levels in the (V)LDL and HDL precipitates were determined by multiplex immunoassay and normalized to the original volumes of plasma. *n* = 20, *** *p* < 0.001 compared to the respective protein levels in the HDL precipitate. Bars represent mean ± standard error of mean (S.E.M).

Four proteins present in the IPA coagulation system pathway were selected for verification using a bead-based immunoassay: von Willebrand factor (VWF), SerpinC1, plasminogen (PLG), and SerpinF2. The selection of these four proteins was based on the availability of antibodies and recombinant proteins needed for the assay. The protein levels of VWF, SerpinC1, PLG, and SerpinF2 were significantly higher in the (V)LDL precipitate compared to the HDL precipitate (Figure 1). 

### 2.2. Bilayer EVs Co-Precipitate with Lipoprotein Particles

**Figure 2:**
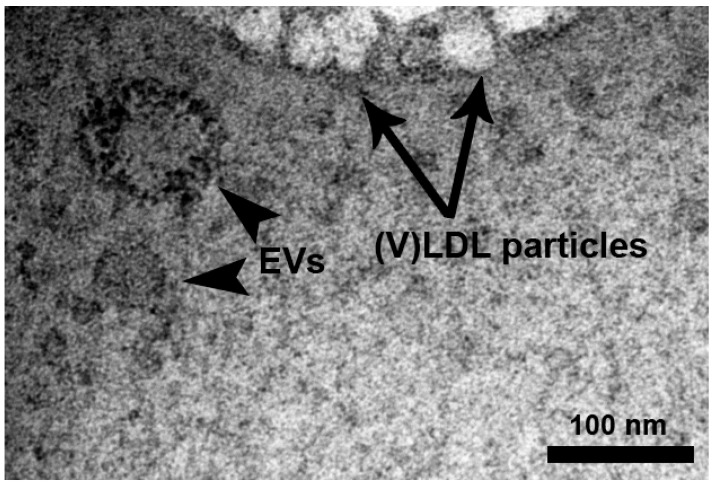
The presence of extracellular vesicles (EVs) in the (V)LDL precipitate. (V)LDL precipitate was processed for electron microscopy as described in the Materials and Methods section. Black arrows indicate bilayer EVs of 60–100 nm in diameter. The blank arrow indicates monolayer lipoprotein particles. Scale bar = 100 nm.

Since EVs have been reported to contain coagulation proteins [5,7], we suspected that plasma EVs might be concomitantly precipitated with (V)LDL particles by dextran sulphate (DS). As expected, monolayer lipoprotein particles with distinct characteristics under the electron microscope (EM) [22,23] were present in the (V)LDL precipitate (Figure 2). In addition, bilayer membrane vesicle-like structures were observed in the plasma (V)LDL precipitate under EM (Figure 2). Those structures showed clear lipid bilayers at a magnification of 87,000× and had a size of 60–100 nm in diameter, identifying the presence of EVs in the (V)LDL precipitate.

### 2.3. Coagulation Proteins in the (V)LDL Precipitate are Present in Extracellular Vesicles (EVs)

**Figure 3:**
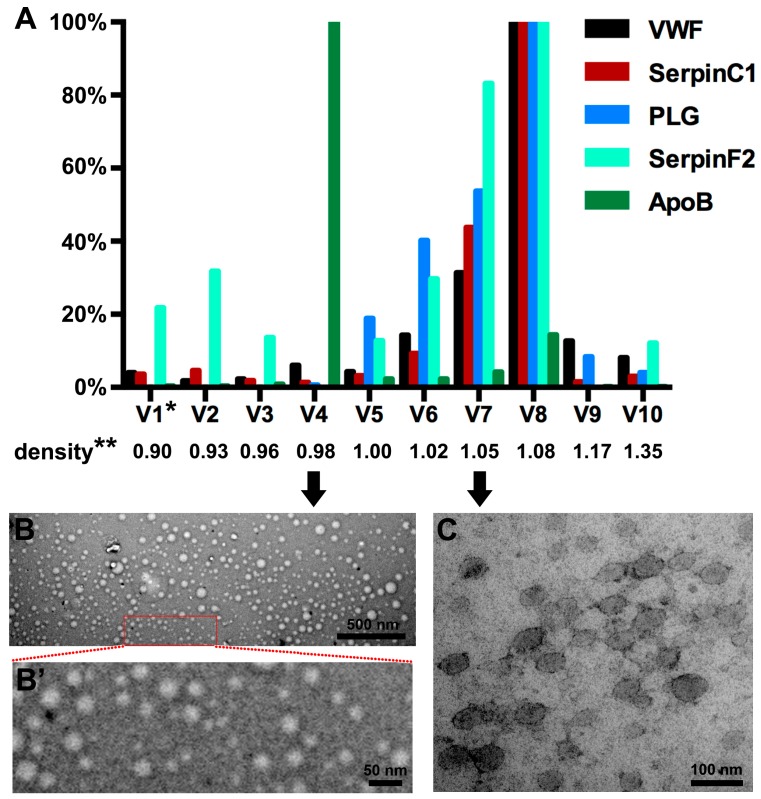
Distribution of VWF, SerpinC1, PLG, SerpinF2, and ApoB in density sub-fractions of the (V)LDL precipitate after density gradient centrifugation. * V1, the first sub-fraction of (V)LDL precipitate. The density of each sub-fraction was indicated accordingly. ** The unit of density is g/mL. (**A**) Protein levels of VWF, SerpinC1, PLG, and SerpinF2 are presented as the percentage of the 8th sub-fraction (V8). Protein levels of ApoB are presented as the percentage of the 4th sub-fraction (V4). (**B**) A representative electron microscope (EM) image of the 4th sub-fraction shows lipoprotein particles. Scale bar = 500 nm. (**B’**) Larger magnification of insert in (**B**). Scale bar = 50 nm. (**C**) A representative EM image of the 7th sub-fraction shows EVs. Scale bar = 100 nm.

Having shown that coagulation pathways and four coagulation proteins were over-represented in the (V)LDL precipitate that contains EVs (Figure 1 and Figure 2), we further investigated whether these coagulation proteins were present in EVs or in lipoprotein particles. For this, we separated lipoprotein particles from EVs using density gradient centrifugation. Lipoprotein particles were enriched in the 4th sub-fraction (density 0.98 g/mL) as shown by the presence of the (V)LDL marker apolipoprotein B (ApoB) protein [24] (Figure 3A). The four selected coagulation pathway proteins VWF, SerpinC1, PLG, and SerpinF2 were mainly present in the 6th–8th sub-fractions (density 1.02–1.08 g/mL) (Figure 3A). Enrichment of (V)LDL particles and EVs in different sub-fractions was confirmed by EM, which showed (V)LDL particles in the 4th sub-fraction (Figure 3B,B’) and bilayer EVs in the 6th–8th sub-fractions (Figure 3C). Abundance of EVs in the 6th–8th sub-fractions was further confirmed by Western blotting for a canonical EV marker, the cluster of differentiation molecule 9 (CD9) [25,26,27] (Figure S1).

### 2.4. Procoagulant Activity

**Figure 4:**
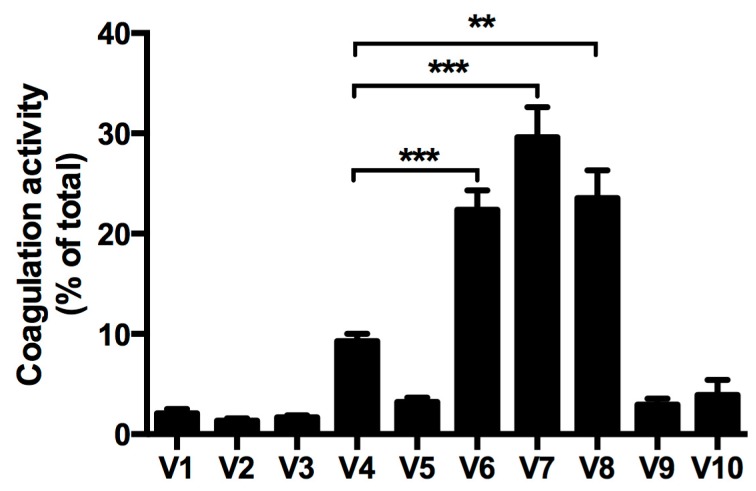
Procoagulant activity in the density gradient sub-fractions of the (V)LDL precipitate. V1, the first sub-fraction of (V)LDL precipitate separated by density gradient centrifugation. The coagulation activity of each sub-fraction was determined by Factor Xa generation and expressed as percentage relative to total coagulation activity. Four independent experiments were performed. Bars represent mean ± S.E.M. Student’s *t*-test compared to V4, ** *p* < 0.01, *** *p* < 0.001.

Since coagulation pathway proteins were mainly present in the EV-rich 6th–8th sub-fractions of (V)LDL precipitate after density gradient centrifugation, we measured the procoagulant activity of each sub-fraction. Among the 10 sub-fractions, the 6th–8th sub-fractions contained abundant EVs and had the highest procoagulant activity (Figure 4 and Table 2). In contrast, the 4th sub-fraction that was enriched with (V)LDL particles showed very low procoagulant activity (less than 10% of total procoagulant activity in the precipitate, Figure 4).

## 3. Discussion

Coagulation and lipid metabolism are two prominent processes involved in the progression of atherosclerosis leading to cardiovascular events such as myocardial infarction and stroke. Plasma EVs have been reported to contain coagulation proteins [5,7] and participate in blood coagulation [28]. We now show that plasma EVs co-precipitated with (V)LDL particles by dextran sulphate (DS) are enriched with coagulation proteins and have procoagulant activity.

LDL apheresis, a well-established lipid-lowering therapy, has been known for decades to reduce LDL as well as coagulation factors [16,17,18,19]. DSA, one of the most widely used methods of LDL apheresis, acutely reduces coagulation factors, such as factor V, VIII, and XI [17,29]. A recent study reported that a population of EVs (200–250 nm) was reduced in plasma after apheresis [8]. However, the EVs depleted by apheresis have not been isolated and characterized. For this, it remains unclear if coagulation proteins are associated with DS-precipitated (V)LDL particles or plasma EVs, or are directly bound to DS. To address these questions, we obtained (V)LDL precipitate from plasma by using DS to mimic DSA apheresis in vitro. In line with DSA LDL apheresis [17,29], coagulation pathway proteins were significantly over-represented in the (V)LDL precipitate compared to HDL precipitate. An EM revealed that the (V)LDL precipitate contained both lipoprotein particles and EVs. Distinct from (V)LDL particles that have a monolayer lipid membrane [22,23], the EVs co-precipitated with (V)LDL particles have a bilayer-membrane and express CD9 [25,26,27,30] (Figure 2, Figure 3 and Figure S1).

Density gradient centrifugation separating (V)LDL particles from EVs showed that the selected four coagulation pathway proteins (VWF, SerpinC1, PLG, and SerpinF2) were present in EVs (Figure 3). This strongly suggests that the removal of coagulation factors by LDL apheresis was due to the removal of EVs from the blood. This is in agreement with the finding that the four coagulation-related proteins (VWF, SerpinC1, PLG, and SerpinF2) identified in the (V)LDL co-precipitated EVs were also retained in the DSA cellulose column during apheresis in severe hypercholesterolemic patients [17]. Furthermore, the procoagulant activity was predominantly detected in the (V)LDL co-precipitated EVs (Figure 4), in line with the higher thrombin generation potential of smaller EVs [8]. A minor part of the coagulation proteins and procoagulant activity were associated with the ApoB-positive (V)LDL fraction (Figure 3 and Figure 4 and Table 2). For this, we cannot rule out that some of the coagulation proteins might be associated with circulating (V)LDL particles. The marginal procoagulant activity of the (V)LDL sub-fraction is presumably due to the lipid membrane that provides a surface for coagulation machinery [31].

**Figure 5:**
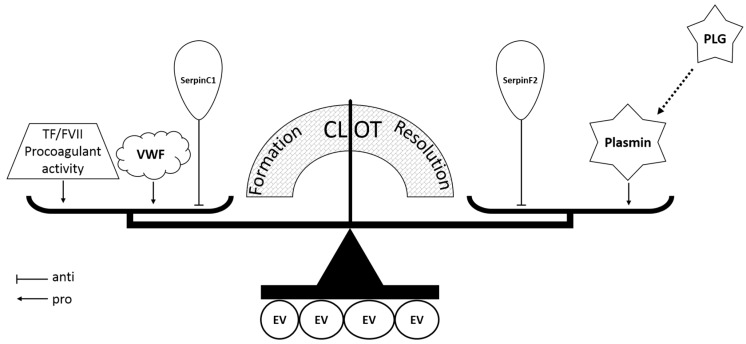
Hypothetical diagram of the coagulation balance on or in the (V)LDL co-precipitated EVs. TF, tissue factor; FVII, coagulation factor VII; VWF, von Willebrand factor; PLG, plasminogen; EV, extracellular vesicle.

Coagulation, anti-coagulation, and fibrinolysis systems are highly regulated and interact to maintain physiological hemostasis. EVs co-precipitated with (V)LDL particles host all three systems regulating the formation and resolution of blood clot (Figure 5). EVs are enriched with coagulation proteins such as VWF, which plays a major role in coagulation by carrying coagulation factor VIII to prevent its premature clearance and recruiting platelets from the circulation to form a hemostatic plug at the damaged site of a blood vessel. Defects in VWF could lead to bleeding disorders [32,33]. SerpinC1, also known as anti-thrombin, plays an important role in anti-coagulation by inhibiting thrombin, factor Xa, and factor IXa [34]. PLG, the inactive form of plasmin that is important in fibrinolysis, can be activated by tissue-type plasminogen activator (tPA) and urokinase-type plasminogen activator (uPA) [35]. SerpinF2, also known as α2-antiplasmin, is a specific inhibitor of plasmin [35]. As plasmin promotes the degradation of the hemostatic clots, the abundance of PLG and SerpinF2 suggests an anti-fibrinolytic property of EVs.

The prominent procoagulant activity (Figure 4) in the EV fractions suggests that the EVs co-precipitated with (V)LDL particles may contain coagulation factor VII and tissue factor. This would be similar to plasma EVs in sepsis and cancer patients that have been shown to contain coagulation factor VII and tissue factor [6,36]. Next to this, EVs have a negatively charged lipid membrane that might serve as a surface for assembling the coagulation machinery [31]. Given that determination of the origin of EVs is challenging [26,30], the origin of (V)LDL co-precipitated EVs was not investigated in the current study. In light of their prominent procoagulant activity, we suspect that (V)LDL co-precipitated EVs are likely derived from endothelial cells and/or monocytes [1,30,37].

The co-precipitation of EVs and lipoprotein particles by dextran sulphate, a negatively charged polysaccharide, points to an unknown shared property by the two distinct membrane vesicles. Given the content and procoagulant activity of (V)LDL co-precipitated EVs, one may postulate that concomitant removal of those EVs during apheresis may contribute to the clinical benefits of apheresis beyond lipid lowering [8]. Apheresis, however, is only used in a small group of patients with severe hypercholesterolemia [11,12,13,14,15]. Statins, a class of drugs that reduce (V)LDL by inhibiting the production of cholesterol, are now also considered as antithrombotic drugs [38]. It is therefore tempting to propose that the antithrombotic pleiotropic effect of statins might be attributed to the modulation of EVs. Further research, however, is needed to evaluate if statin therapy would also affect coagulation protein content and coagulation activity in plasma EVs.

A limitation of this study is that since only healthy subjects were enrolled, the pathophysiological and biomarker roles of (V)LDL co-precipitated EVs in cardiovascular disease [1] remain unclear. Studies on patients subjected to LDL apheresis are therefore needed. A better understanding of how (V)LDL co-precipitated EVs impacts the balance of pro- and anti-coagulation in various organ systems may lead to the design of molecular interventions via EVs to prevent thrombosis or control bleeding. Furthermore, following the example of cancer therapeutics [39], personalized medicine approaches based on (V)LDL co-precipitated EVs may allow for the assessment of susceptibility to coagulation from quantifying EV content, or allow the design of optimal EV-content for therapeutically attenuating these processes.

## 4. Materials and Methods

### 4.1. Study Subjects and Sample Preparation

Plasma from 20 healthy volunteers (10 male and 10 female, average age 30.3 ± 8.2 years) was used for this study. Blood was collected into ethylenediaminetetraacetic acid (EDTA) tubes and centrifuged at 2000 *g* for 10 min at 4 °C as previously described [40]. The plasma was collected and stored at −80 °C for further analysis. This study was approved by the institutional review board (IRB, institutional ethics committee, B-15-094, 18 September 2015) of the National University of Singapore, and written informed consent was obtained from all participants according to the Declaration of Helsinki.

### 4.2. Sequential Precipitation of (V)LDL Fraction and HDL Fraction

(V)LDL and HDL fractions were selectively precipitated as previously described [40,41]. Briefly, 6.5% (*w/v*) dextran sulphate (DS) and 2 M Manganese (II) chloride (MnCl_2_) (Sigma-Aldrich, St. Louis, MO, USA) stock solutions were prepared in Milli-Q H_2_O. To precipitate (V)LDL fraction, DS stock solution (1:125) and MnCl_2_ stock solution (1:40) were added to plasma. The plasma sample was vortexed and centrifuged at 4800 *g* for 10 min at 4 °C. The pellet was collected as (V)LDL fraction and the supernatant was transferred to a new tube. DS stock solution (1:10) and MnCl_2_ stock solution (1:10) were added to the supernatant. The sample was vortexed and then incubated for 2 h at 4 °C. After incubation, the HDL fraction was isolated by centrifugation at 4800 *g* for 10 min at 4 °C.

### 4.3. Proteomics and IPA Analysis

Liquid chromatography-tandem mass spectrometry (LC-MS/MS) was used for label-free proteomic analysis as described [42]. Briefly, LC-MS/MS (Agilent Technologies Inc., Santa Clara, CA, USA) was used to analyze purified proteins from the (V)LDL and HDL precipitates. The two precipitates (approximately 50 μg each) were resolved in two lanes of a 10% SDS-PAGE gel (Bio-Rad, Hercules, CA, USA), respectively, and subsequently stained with Coomassie blue. Each lane was excised after gel destaining and divided into six pieces equally for in-gel trypsin digestion; after reduction with 20 mm dithiothreitol (DTT) and alkylation using 55 mm iodoacetamide (IAA), each piece was digested with sequencing-grade trypsin (Promega, Madison, WI, USA) overnight. Digested peptides were extracted from the gel, dried, and then desalted. The peptides were analyzed with a Q-Exactive mass spectrometer (Thermo Scientific, San Jose, CA, USA) coupled with an Ultimate 3000 RSLC nano-HPLC system (Dionex, Amsterdam, The Netherlands). The raw data were converted to mascot generic (mgf) files using Proteome Discoverer ver. 1.4 (Thermo Scientific, San Jose, CA, USA), and then the mgf files were searched against the Uniprot human proteome database (Released on 29 November 2013; 176,946 sequences, 70,141,034 residues) using an in-house Mascot Server ver. 2.4.1 (Matrix Science, London, UK).

Ingenuity Pathway Analysis (IPA^®^, QIAGEN, Redwood City, CA, USA, Build Version 321501M, Content Version 17199142) was used to determine canonical pathways statistically over-represented in the proteins that were only in the (V)LDL precipitate. Proteins of pathways significantly over-represented were selected for verification based on antibody and recombinant protein availability to set up the multiplex immunoassay.

### 4.4. Quantitative Measurement of Protein Levels

Protein levels were determined using a bead-based immunoassay as previously described [3]. In short, the beads (Luminex #MagPlex-C Microspheres, MC100-xx, Austin, TX, USA) were conjugated with selected antibodies to form a bead-capture antibody complex. Samples were incubated with the bead-capture antibody complex followed by the biotinylated antibodies. Streptavidin-phycoerythrin (SA-PE, BD Bioscience #554061, San Jose, CA, USA) was added to quantify the concentration of captured proteins. Standard dilution curves for homologous recombinant proteins were correlated to the SA-PE signal. Data were acquired and analyzed with the Bio-Plex^®^ 200 Systems (Bio-Rad #171-000201, Hercules, CA, USA). The antibodies and recombinant proteins were listed as follows. Detection of von Willebrand factor (VWF) used recombinant human VWF protein (Factor VIII free, Fitzgerald #30C-CP4003U, Fitzgerald Industries International, Acton, MA, USA), anti-human VWF (Fitzgerald #70R-10589, Fitzgerald Industries International, Acton, MA, USA), and biotinylated anti-human VWF (Fitzgerald #60R-1019, Fitzgerald Industries International, Acton, MA, USA); detection of SerpinC1 used antithrombin III antibody (NOVUS Biologicals #NBP1-05149, Littleton, CO, USA), human SerpinC1 biotinylated affinity purified antibody (R&D Systems #BAF1267, Minneapolis, MN, USA), and recombinant human SerpinC1 (R&D Systems #1267-PI-010, Minneapolis, MN, USA); detection of plasminogen used anti-human plasminogen (HyTest #4P11, Turku, Finland), biotinylated anti-human plasminogen (HyTest #4P11B, Turku, Finland), and recombinant human plasminogen (BBI Solutions #P204-1; Cardiff, UK); and detection of SerpinF2 used anti-human SerpinF2 (R&D Systems #MAB1470, Minneapolis, MN, USA), biotinylated anti-human SerpinF2 (R&D Systems #BAF1470, Minneapolis, MN, USA), and recombinant human SerpinF2 (R&D Systems #1470-PI-010, Minneapolis, MN, USA). This assay is referred to as a multiplex immunoassay.

### 4.5. Apolipoprotein B Measurement

Apolipoprotein B (ApoB) was measured in triplicate with an ELISA kit (R&D Systems #DAPB00, Minneapolis, MN, USA) according to the manufacturer’s instructions.

### 4.6. Density Gradient Centrifugation

(V)LDL precipitate was isolated from 8 mL plasma as described above. In order to make the density gradient buffer, five solutions containing different concentrations of OptiPrepTM medium (OptiPrep, Axis-shiled #1114542, Oslo, Norway) were prepared with 10× PBS pH 7.4 (Ambion^®^, Life Technologies #AM9265, Carlsbad, CA, USA) and Milli-Q H_2_O. The working solutions comprised 5%, 10%, 20%, 30%, and 40% OptiPrep and 1× PBS respectively. The solutions were added and overlaid into the ultracentrifuge tubes (Beckman Coulter #344059, Brea, CA, USA) sequentially with the highest density (40% OptiPrep) on the bottom. Subsequently, the isolated (V)LDL precipitate was carefully placed on top of the density gradient buffer followed by centrifugation at 200,000 *g* for 18 h at 4 °C with low acceleration and low brake (Beckman Coulter #Optima XL-90 Ultracentrifuge #SW 41 Ti Rotor, Brea, CA, USA). Afterwards, 10 different gradient sub-fractions (1 mL per sub-fraction) were harvested sequentially (from top to bottom) and transferred into 1.5-mL Eppendorf tubes.

After thorough vortexing, 900 µL of each sub-fraction was transferred into a new ultracentrifuge tube, respectively, containing 7 mL 0.1% BSA buffer (Sigma-Aldrich #05470, in 1× PBS, m/v, St. Louis, MO, USA), and centrifuged at 200,000 *g* for 1 h at 4 °C. The pellets were resuspended in 200 µL lysis buffer (Roche #04719956001, Basel, Switzerland) for protein level measurement or in 200 µL 1× PBS for procoagulant activity assay. The remaining 100 µL of the sub-fractions were used for density measurement and electron microscopy.

### 4.7. Electron Microscopy

Both the (V)LDL precipitate (resuspended in 1× PBS) and its sub-fractions from density gradient centrifugation (100 µL leftover of each sub-fraction) were processed at room temperature (20 °C) for electron microscopy (EM). The samples were diluted 1:5 in 1× PBS prior to fixation with 2.0% glutaraldehyde (Sigma-Aldrich #G5882, St. Louis, MO, USA). After fixation, a 75-mesh grid (Agar scientific #G2075C, Stansted, UK) was laid on a drop of sample for 10 min; then, the grid was rinsed 10 times with Milli-Q H_2_O (1 min per rinse). For staining, the grid was firstly laid on a drop of uranyl acetate (pH 7.0, SPI-CHEM #2624, West Chester, PA, USA) for 10 min. The grid was subsequently rinsed with Milli-Q H_2_O and methylcellulose uranyl (pH 4.0), and then incubated for 10 min on a drop of methylcellulose uranyl (pH 4.0, Sigma-Aldrich #M-6385, St. Louis, MO, USA). The samples were analyzed with an FEI Tecnai™ T12 electron microscope (FEI Company, Eindhoven, The Netherlands).

### 4.8. EV Associated Procoagulant Activity Assay

Sub-fractions of (V)LDL precipitate obtained by density gradient centrifugation were resuspended in 200 µL PBS. EV-associated procoagulant activity was measured as previously described with adaptations [43,44]. Briefly, 20 µL of each sub-fraction in PBS was incubated for 15 min in 100 µL incubation buffer containing 10 mM HEPES, 25 µM negatively charged phospholipids (dioleoylphosphatidylserine and dioleoylphosphatidylcholine at 1:9, Avanti Polar Lipids #840035P, #850375P, Alabaster, AL, USA), 137 mM NaCl, 4 mM KCl, 6 mM CaCl_2_, 5 mg/mL bovine serum albumin, and 5 units/mL hirudin (Sigma-Aldrich #H0393, pH 7.45, St. Louis, MO, USA). Subsequently, 40 µL 5.63 nM Factor VII (Enzyme Research Laboratories #HFVII1007, South Bend, IN, USA) or incubation buffer was added and the mixture was incubated at room temperature for 10 min. After incubation, 25 µL Factor Xa chromogenic substrate S2765 (Chromogenix #82141339, Werfen, Barcelona, Spain) was added followed by 40 µL of 281 nM Factor X (Enzyme Research Laboratories #HFX1010, South Bend, IN, USA) to start the reaction. The rate of the chromophoric group p-nitroaniline formation was recorded for 90 min at 405 nm and the rate of Factor Xa generation was calculated. Procoagulant activity was expressed as the rate of Factor Xa generation in fM Xa/min. Recombinant human tissue factor (Dade^®^ Innovin, Siemens Healthcare Diagnostics #B4212, Tarrytown, NY, USA) was used as a positive control.

### 4.9. Statistics

EV protein levels in the (V)LDL precipitate and HDL precipitate were analyzed using a paired t-test as each pair of (V)LDL and HDL fractions was from individual subjects. All statistical analysis was performed using SPSS^®^ (IBM^®^, Version 22.0.0.0; Armonk, NY, USA).

## 5. Conclusions

Procoagulant EVs are concomitantly precipitated with (V)LDL particles from plasma by dextran sulphate used in LDL apheresis. Our findings suggest that a loss of coagulation factors and coagulation activity during LDL apheresis may be explained by the concomitant removal of procoagulant EVs and (V)LDL particles.

## Figures and Tables

**Table 1:** Canonical Pathways over-represented in the very-low- and low-density lipoprotein ((V)LDL) precipitate.

Canonical Pathway	−Log (*p*-Value)	
	(V)LDL	HDL
Coagulation System	10.26	2.93
Extrinsic Prothrombin Activation	7.34	2.39
Intrinsic Prothrombin Activation	6.01	1.92

HDL: high-density lipoprotein.

**Table 2:** Procoagulant activity in the density gradient sub-fractions of the (V)LDL precipitate.

Sub-Fraction of (V)LDL Precipitate	Factor Xa Generation (fM Xa/min)
V1	145.67 ± 44.98
V2	101.17 ± 30.21
V3	124.58 ± 33.16
V4	673.75 ± 153.93
V5	239.67 ± 57.03
V6	1592.17 ± 304.77 *
V7	2028.33 ± 324.72 **
V8	1596.83 ± 272.50 *
V9	223.25 ± 72.88
V10	309.42 ± 148.90
Total	7034.83 ± 1237.64

Data are presented as mean ± S.E.M. Student’s *t*-test compared to V4, * *p* <0.05, ** *p* <0.01.

## Supplementary Materials

Supplementary materials can be found at www.mdpi.com/1422-0067/19/1/94/s1.

## Abbreviations

(V)LDL	Very-low- and low- density lipoproteins
DSA	Dextran sulphate adsorption
EV	Extracellular vesicle
HDL	High-density lipoprotein
LC-MS/MS	Liquid chromatography-tandem mass spectrometry
IPA	Ingenuity Pathway Analysis
SA-PE	Streptavidin-phycoerythrin
VWF	von Willebrand factor
ELISA	Enzyme-linked immunosorbent assay
ApoB	Apolipoprotein B
BSA	Bovine serum albumin
PBS	Phosphate-buffered saline
TF	Tissue factor
EM	Electron Microscopy
PLG	Plasminogen
tPA	Tissue-type plasminogen activator
uPA	Urokinase-type plasminogen activator
