# Supplementary material for: Lowering Low-Density Lipoprotein Particles in Plasma Using Dextran Sulphate Co-Precipitates Procoagulant Extracellular Vesicles
Source: Int J Mol Sci. 2017 Dec 29;19(1):94. doi: 10.3390/ijms19010094 (PMC5796044; doi:10.3390/ijms19010094)
Supplement: Supplementary file 1 [file ijms-19-00094-s001.pdf]

Supplemental Table 1 Proteins only present in VLDL fraction

	Entrez Gene Name	Coagulation System	Extrinsic Prothrombin Activation	Intrinsic Prothrombin Activation	Cellular location
1	ADP-ribosylation factor interacting protein 1	-	-	-	Cytoplasm
2	afamin	-	-	-	Extracellular
3	alpha-1-B glycoprotein	-	-	-	Extracellular
4	alpha-2-macroglobulin	+	-	-	Extracellular
5	alpha-2-macroglobulin-like 1	-	-	-	Cytoplasm
6	angiotensinogen (serpin peptidase inhibitor, clade A, member 8)	-	-	-	Extracellular
7	angiotensinogen (serpin peptidase inhibitor, clade A, member 8)	-	-	-	Extracellular
8	apolipoprotein B	-	-	-	Extracellular
9	apolipoprotein B	-	-	-	Extracellular
10	apolipoprotein E	-	-	-	Extracellular
11	apolipoprotein E	-	-	-	Extracellular
12	apolipoprotein E	-	-	-	Extracellular
13	ArfGAP with GTPase domain, ankyrin repeat and PH domain 6	-	-	-	Other
14	ATP-binding cassette, sub-family A (ABC1), member 1	-	-	-	Plasma Membrane
15	ATP-binding cassette, sub-family C (CFTR/MRP), member 12	-	-	-	Cytoplasm
16	attractin	-	-	-	Extracellular
17	Bardet-Biedl syndrome 4	-	-	-	Cytoplasm
18	carboxypeptidase N, polypeptide 2	-	-	-	Extracellular
19	caspase 8, apoptosis-related cysteine peptidase	-	-	-	Nucleus
20	Cbl proto-oncogene, E3 ubiquitin protein ligase	-	-	-	Nucleus
21	ceruloplasmin (ferroxidase)	-	-	-	Extracellular
22	chemokine (C-X-C motif) ligand 2	-	-	-	Extracellular
23	cholinergic receptor, muscarinic 2	-	-	-	Plasma

					Membrane
24	chromosome 6 open reading frame 163	-	-	-	Other
25	clathrin, heavy chain (Hc)	-	-	-	Plasma Membrane
26	coagulation factor V (proaccelerin, labile factor)	+	+	+	Plasma Membrane
27	coagulation factor XII (Hageman factor)	+	+	+	Extracellular
28	coagulation factor XIII, A1 polypeptide	+	+	+	Extracellular
29	coagulation factor XIII, B polypeptide	+	+	+	Extracellular
30	collagen, type IV, alpha 3 (Goodpasture antigen) binding protein	-	-	-	Cytoplasm
31	collagen, type IX, alpha 3	-	-	-	Extracellular
32	complement component 2	-	-	-	Extracellular
33	complement component 3	-	-	-	Extracellular
34	complement component 8, alpha polypeptide	-	-	-	Extracellular
35	complement factor B	-	-	-	Extracellular
36	cornulin	-	-	-	Cytoplasm
37	cyclin and CBS domain divalent metal cation transport mediator 2	-	-	-	Plasma Membrane
38	diaphanous-related formin 2	-	-	-	Cytoplasm
39	dimethylarginine dimethylaminohydrolase 1	-	-	-	Cytoplasm
40	endoglin	-	-	-	Plasma Membrane
41	envoplakin	-	-	-	Plasma Membrane
42	EPS8-like 2	-	-	-	Other
43	exocyst complex component 5	-	-	-	Cytoplasm
44	extracellular matrix protein 1	-	-	-	Extracellular
45	family with sequence similarity 186, member A	-	-	-	Other
46	F-box and leucine-rich repeat protein 19	-	-	-	Other
47	fibrinogen alpha chain	+	+	+	Extracellular

48	fibronectin 1	-	-	-	Extracellular
49	ficolin (collagen/fibrinogen domain containing) 3	-	-	-	Extracellular
50	flightless I homolog (Drosophila)	-	-	-	Nucleus
51	flightless I homolog (Drosophila)	-	-	-	Nucleus
52	gelsolin	-	-	-	Extracellular
53	growth factor receptor-bound protein 2	-	-	-	Cytoplasm
54	histone cluster 1, H1b	-	-	-	Nucleus
55	hyaluronan binding protein 2	-	-	-	Extracellular
56	immunoglobulin heavy constant gamma 1 (G1m marker)	-	-	-	Extracellular
57	immunoglobulin heavy constant gamma 1 (G1m marker)	-	-	-	Extracellular
58	immunoglobulin heavy constant gamma 1 (G1m marker)	-	-	-	Extracellular
59	immunoglobulin heavy constant mu	-	-	-	Plasma Membrane
60	immunoglobulin heavy locus	-	-	-	Other
61	immunoglobulin J polypeptide, linker protein for immunoglobulin alpha and mu polypeptides	-	-	-	Extracellular
62	immunoglobulin kappa constant	-	-	-	Extracellular
63	immunoglobulin kappa variable 3-20	-	-	-	Extracellular
64	immunoglobulin lambda constant 1 (Mcg marker)	-	-	-	Cytoplasm
65	immunoglobulin lambda constant 1 (Mcg marker)	-	-	-	Cytoplasm
66	immunoglobulin lambda constant 1 (Mcg marker)	-	-	-	Cytoplasm
67	insulin receptor	-	-	-	Plasma Membrane
68	insulin-like growth factor binding protein, acid labile subunit	-	-	-	Extracellular
69	inter-alpha-trypsin inhibitor heavy chain 1	-	-	-	Extracellular
70	inter-alpha-trypsin inhibitor heavy chain 3	-	-	-	Extracellular
71	IQ motif containing E	-	-	-	Cytoplasm
72	junction plakoglobin	-	-	-	Plasma Membrane
73	keratin 1	-	-	-	Cytoplasm

74	keratin 10	-	-	-	Cytoplasm
75	keratin 15	-	-	-	Cytoplasm
76	keratin 18	-	-	-	Cytoplasm
77	keratin 19	-	-	-	Cytoplasm
78	keratin 2	-	-	-	Other
79	keratin 24	-	-	-	Cytoplasm
80	keratin 25	-	-	-	Other
81	keratin 27	-	-	-	Other
82	keratin 35	-	-	-	Cytoplasm
83	keratin 38	-	-	-	Cytoplasm
84	keratin 6B	-	-	-	Cytoplasm
85	keratin 76	-	-	-	Cytoplasm
86	keratin 77	-	-	-	Cytoplasm
87	keratin 9	-	-	-	Other
88	kinesin family member 1B	-	-	-	Cytoplasm
89	kinesin family member 20B	-	-	-	Nucleus
90	kinesin family member 3C	-	-	-	Cytoplasm
91	Kirsten rat sarcoma viral oncogene homolog	-	-	-	Cytoplasm
92	leucine rich repeat containing 48	-	-	-	Cytoplasm
93	lipoprotein, Lp(a)	-	-	-	Extracellular
94	microcephalin 1	-	-	-	Nucleus
95	minichromosome maintenance complex component 10	-	-	-	Nucleus
96	MIR7-3 host gene (non-protein coding)	-	-	-	Other
97	mitotic spindle organizing protein 2B	-	-	-	Cytoplasm
98	multiple PDZ domain protein	-	-	-	Plasma Membrane
99	myelin expression factor 2	-	-	-	Nucleus
100	myosin VA (heavy chain 12, myoxin)	-	-	-	Cytoplasm
101	NEDD4 binding protein 2	-	-	-	Cytoplasm
102	nicotinate phosphoribosyltransferase	-	-	-	Cytoplasm

103	nipsnap homolog 1 (C. elegans)	-	-	-	Cytoplasm
104	olfactomedin 3	-	-	-	Cytoplasm
105	phosphoglycerate kinase 2	-	-	-	Cytoplasm
106	phospholipase D family, member 5	-	-	-	Other
107	phospholipase D family, member 5	-	-	-	Other
108	phosphoprotein membrane anchor with glycosphingolipid microdomains 1	-	-	-	Plasma Membrane
109	phosphorylase kinase, beta	-	-	-	Cytoplasm
110	protein tyrosine phosphatase, receptor type, G	-	-	-	Plasma Membrane
111	protocadherin gamma subfamily C, 5	-	-	-	Cytoplasm
112	Ras association (RalGDS/AF-6) domain family member 6	-	-	-	Other
113	RB1-inducible coiled-coil 1	-	-	-	Nucleus
114	regulator of G-protein signaling 7	-	-	-	Cytoplasm
115	Rho GTPase activating protein 36	-	-	-	Cytoplasm
116	ribosomal protein L32	-	-	-	Cytoplasm
117	serpin peptidase inhibitor, clade A (alpha-1 antiproteinase, antitrypsin), member 7	-	-	-	Extracellular
118	serpin peptidase inhibitor, clade F (alpha-2 antiplasmin, pigment epithelium derived factor), member 2	+	-	-	Extracellular
119	SH2B adaptor protein 3	-	-	-	Plasma Membrane
120	SH3 and multiple ankyrin repeat domains 2	-	-	-	Plasma Membrane
121	SHC SH2-domain binding protein 1-like	-	-	-	Other
122	Sp4 transcription factor	-	-	-	Nucleus
123	spectrin repeat containing, nuclear envelope 1	-	-	-	Nucleus
124	spectrin repeat containing, nuclear envelope 2	-	-	-	Nucleus
125	spectrin repeat containing, nuclear envelope 2	-	-	-	Nucleus
126	sphingosine-1-phosphate phosphatase 2	-	-	-	Cytoplasm

127	ST3 beta-galactoside alpha-2,3-sialyltransferase 6	-	-	-	Cytoplasm
128	sterile alpha motif domain containing 11	-	-	-	Nucleus
129	synovial sarcoma, X breakpoint 5	-	-	-	Other
130	TAF15 RNA polymerase II, TATA box binding protein (TBP)-associated factor, 68kDa	-	-	-	Nucleus
131	TATA box binding protein (TBP)-associated factor, RNA polymerase I, A	-	-	-	Nucleus
132	tetratricopeptide repeat domain 16	-	-	-	Other
133	thyroid hormone receptor interactor 12	-	-	-	Cytoplasm
134	thyroid hormone receptor interactor 6	-	-	-	Extracellular
135	titin	-	-	-	Cytoplasm
136	transmembrane phosphoinositide 3-phosphatase and tensin homolog 2 pseudogene 1	-	-	-	Other
137	transmembrane protein 131	-	-	-	Extracellular
138	tudor domain containing 15	-	-	-	Other
139	ubiquitously transcribed tetratricopeptide repeat containing, Y-linked	-	-	-	Other
140	ubiquitously transcribed tetratricopeptide repeat containing, Y-linked	-	-	-	Other
141	uroporphyrinogen decarboxylase	-	-	-	Cytoplasm
142	v-myb avian myeloblastosis viral oncogene homolog	-	-	-	Nucleus
143	von Willebrand factor	+	-	-	Extracellular
144	zinc finger protein 227	-	-	-	Nucleus

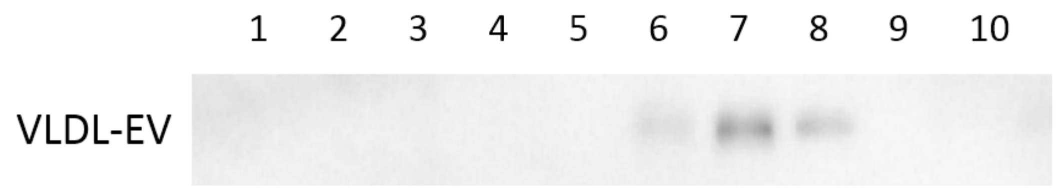

Supplemental Figure S1. Distribution of CD9 in (V)LDL sub-fractions after density gradient centrifugation.

Numbers on top stand for the sub-fractions after density gradient experiments. The sub-fractions were the same ones as in Figure 3. The sub-fractions after density gradient experiment were pooled respectively from 20 healthy donors and lysed with 0.1% Sodium dodecyl sulfate (SDS) for Western Blot. Western Blot was performed on the 4-12% gradient Bis-Trisgel (NuPage, Invitrogen). The antibodies used in Western Blot for CD9: CD9 Antibody (C-4) (santa cruz biotechnology#SC13118, primary antibody), Polyclonal Goat Anti-Mouse Immunoglobulins (Dako# P0447, secondary antibody). For visualization, the ECL kit HRP (Substrate), Immobilon Western Chemiluminescent HRP substrate (Milipore #WBKLS0500), was used.
